# Stress Among the Adult Population During COVID-19 Pandemic in Kedah, Malaysia: Association Between Sociodemographics and the Movement Control Order Period

**DOI:** 10.7759/cureus.47619

**Published:** 2023-10-25

**Authors:** Amalina Ismail, Tengku A Tengku Ismail, Muhammad F Mohamad Marzuki

**Affiliations:** 1 Department of Community Medicine, Universiti Sains Malaysia, Kubang Kerian, MYS; 2 Non-Communicable Disease Control Unit, Kedah State Health Department, Alor Setar, MYS

**Keywords:** movement control order, covid-19, dass-21, adult, stress

## Abstract

Background

The COVID-19 pandemic has led to a significant increase in the prevalence of stress worldwide. However, the extent and factors associated with psychological distress during COVID-19 among the local population in Kedah, Malaysia, have not been adequately explored. This study aimed to determine the prevalence and factors associated with stress among the adult population in Kedah, Malaysia, during the COVID-19 pandemic.

Materials and methods

We conducted a cross-sectional study using a retrospective record review. A simple random sampling was applied among the adult population of Kedah who were screened for mental health well-being from January 2021 until March 2022. A proforma checklist that consists of sociodemographic and clinical factors and the date of screening was used to collect the data. A descriptive and multiple logistic regression was conducted, and analysis was done by SPSS version 26 (IBM Inc., Armonk, New York). The dependent variables were the presence of stress incorporated self-reported symptoms ranging from mild to very severe. The independent variables were sociodemographic, clinical factors, and movement control order period, which refers to the period when the state of Kedah experienced all the different phases of movement control order (MCO), which were from 1 January 2021 until 28 June 2021.

Results

In total, 562 adults were included. The mean age was 31.75 years, and the majority were female (69.6%). The prevalence of stress was 45.7% (95% CI 41.6%, 49.8%), with a total of 257 people. The majority of them did not have stress, consisting of 305 people (54.3%), followed by 69 people (12.3%) who reported severe stress, 67 people (11.9%) who reported moderate, 66 people (11.7%) who reported mild, and 55 people (9.8%) who reported very severe stress. The significant factor associated with stress among the adult population in Kedah, Malaysia, during the COVID-19 pandemic was the female gender (Adj OR 3.035 95% CI: 2.007 to 4.591, p-value <0.001). Being unemployed (Adj OR 2.171 95% CI: 1.480 to 3.185, p-value <0.001) and being under movement control order period was also associated with stress (Adj OR 0.383 95% CI: 0.264 to 0.555, p-value <0.001).

Conclusion

The prevalence of stress among the adult population during the COVID-19 pandemic in Kedah was 45.7%, with a total of 257 people, higher than other studies. Being female and unemployed was associated with stress, while the movement control order period was a protective factor against stress. Preventive strategies should be tailored based on the vulnerabilities of these groups, such as the development of more effective community-based interventions for safeguarding the mental health of the general public during future pandemics.

## Introduction

The World Health Organization (WHO) defines health as a state of complete physical, mental, and social well-being and not merely the absence of disease or infirmity. The mental well-being of individuals plays a crucial role in their cognitive abilities, emotional expression, social interactions, professional pursuits, and overall life satisfaction [1]. Objective 3.4 of the United Nations Sustainable Development Goals (SDGs) highlights the importance of mental health in assuring a high quality of life [2]. Stress is commonly observed as a response to external stressors or challenges, especially when they are perceived as threatening. It originates from the release of specific hormones within the brain [3] and is one of the important mental health issues worldwide.

On March 12, 2020, WHO designated COVID-19 a global pandemic due to its rapid global spread following its emergence in Wuhan, Hubei Province, China, in December 2019 [4]. It has affected numerous countries, including Malaysia. Since the identification of the first COVID-19 case in Malaysia on January 25, 2020, the virus has caused havoc throughout the country, causing major disruptions and difficulties [4]. The global spread of COVID-19 has significantly increased the prevalence and severity of severe mental health issues. Consistent with prior research conducted in Hong Kong and the United Kingdom, it appears that the general population began to manifest more severe mental health symptoms as the pandemic progressed [5].

The prevalence of clinically significant levels of mental distress in the general population increased from 18.9% in 2018-19 to 27.3% in April 2020, one month into the UK lockdown [6]. In a study conducted among Malaysians aged 18 years and older using an anonymous internet-based Depression, Anxiety, and Stress Scale - 21 Items (DASS-21), the prevalence of stress increased from 11.5% to 30.6% over a 16-week period [5].

Prolonged stress increases the likelihood of other mental health issues, such as anxiety and depression [3]. Failure to treat mental illnesses can result in a number of significant consequences, including strained relationships with friends and family (69.2%), suicidal ideation (56.3%), financial difficulties (51%), and falling out of school or losing a job (50.4%), as well as a decline in academic performance (49%) [7].

Malaysia reported its first three cases of COVID-19 on January 25, 2020. In response to the pandemic, on March 16, 2020, the Malaysian prime minister issued the unprecedented movement control order (MCO). MCO was implemented in Malaysia, mandating that everyone remain at home and permitting only essential services to operate. However, experiencing isolation or limited access to resources has negative effects on mental health, mainly attributable to feelings of boredom, irritation, and financial stress [8]. Stress can negatively impact physical health, increasing susceptibility to COVID-19 infection and exacerbating the disease's severity [9]. It can also influence parental skills, resulting in negative effects on the behavior and mental health of children [10]. It has been discovered that the presence of stress-related disorders, such as post-traumatic stress disorder (PTSD), acute stress reactions, and adjustment disorders following traumatic or stressful life events, is associated with an increased risk of overall mortality and mortality due to preventable unnatural causes. The rising occurrence of stress-related disorders amid the ongoing COVID-19 pandemic offers significant insights that can inform the development of preventive strategies targeted at mitigating the risk of early mortality among individuals who have experienced stress-related disorders [11].

This study aims to determine the prevalence and factors associated with stress among the adult population in Kedah, Malaysia, during the COVID-19 pandemic. Thus, it could play a vital role in the expansion of mental health programs, with a particular emphasis on addressing mental health issues in communities. This ultimately contributes to the well-being of individuals and the population as a whole.

## Materials and methods

Design and setting

The study was a cross-sectional study using a retrospective record review conducted between January 2023 and June 2023. It was conducted among the adult population of Kedah who were screened for mental health well-being in 2021 and 2022. Kedah, which is located in the north of Peninsular Malaysia, had a total land area of 9,492 km*2* in 2020 and a total population of 2.2 million in 2021 [12]. According to the National Health and Morbidity Survey 2015 main report, 30.3% of adults with mental health problems were those who lived in rural area, and the prevalence in Kedah was 26.7% [13].

Kedah residents aged 18 to 60 years who underwent screening between January 2021 and March 2022 were included in this study. Cases involving duplicate data, where there are repeated entries for the same individual in the dataset, have been excluded from the study. Additionally, data related to healthcare professionals has been omitted from the study, as it specifically focuses on the general adult population. The sample size was calculated using a single proportion formula for the prevalence of stress, which was (Zα∕2)/Δ)^2*p(1-p). Power and sample size (PS) software for two proportions formula to determine the associated factors. The biggest sample size was from the first formula. According to Tee et al. [14], 11.6% of unemployed individuals in the Philippines experienced stress. This study employed a required sample size denoted by 'n,' along with a Z statistic value of 1.96 for a 95% confidence interval. The estimated prevalence of stress, represented by 'p,' was considered along with a 5% absolute precision of the margin of error, denoted by 'd.' Taking into consideration a 10% chance of data entry errors, it was determined that a sample size of 562 participants would be sufficient for the study. They were recruited from the adult population of Kedah who fulfilled the inclusion and exclusion criteria and were screened for mental health in 2021 and 2022. Meanwhile, from January 2021 to March 2022, 3988 individuals registered for the online screening of 'Saringan Minda Sihat' (DASS-21). Out of 3988, 1983 of them fulfilled the study criteria. Then simple random sampling was done to obtain 562 out of these 1983 using SPSS version 26 (IBM Inc., Armonk, New York) random number generator.

Participants 

Participants were recruited from the adult population of Kedah who fulfilled the inclusion and exclusion criteria and were screened for mental health in 2021 and 2022. The participants in this screening program completed an online questionnaire, and their responses were thereafter stored in the online database upon questionnaire submission. 

Data source

This study utilized two tools for data collection. These tools included the database of 'Saringan Minda Sihat (DASS-21) and the ProForma checklist. This study utilized the existing 'Saringan Minda Sihat' (DASS-21) database as a secondary data source to collect the required data for this study. The Non-Communicable Disease Unit of Kedah State Health Department manages the database. This mental health screening was established in 2021 and is disseminated to the community via the Facebook pages of the Kedah State Health Department and the District Health Office, as well as during the community health program.

The database consists of sociodemographic data, clinical data, level of depression, anxiety and stress, date of case registered, action plan, interventions, issues that can be the stressor, and referral.

Data for this study was collected via the ProForma checklist. The data required were sociodemographic and clinical factors, the outcome of the screening, and the date of screening to determine whether the screening was done during or after the MCO period. The data were then input into Microsoft Excel (Microsoft, Redmond, Washington) before being exported to SPSS version 26. The Medical Research and Ethics Committee, Ministry of Health (MREC) (NMRR-22-02747-XZA IIR), and Human Research Ethics Committee (USM/JEPeM/22110715) approval for the study were acquired before initiating any study-related activities.

The study had two main outcomes. Using the DASS-21 stress subscale, which classifies stress levels as normal, mild, moderate, severe, or very severe, the study aimed to determine the prevalence of stress among adults. The prevalence was determined by dividing the number of adults experiencing stress by the total number of adults screened in Kedah between January 2021 and March 2022. The second objective was to determine the factors associated with stress. This study's dependent variable was dichotomous, classifying individuals into two groups: those with a presence of stress and those designated "normal" in terms of stress levels. The "presence of stress" incorporated self-reported symptoms ranging from mild to very severe, as measured by a DASS-21 score greater than seven, as documented in the database. Data on sociodemographic and clinical factors, as well as the date of screening, were collected in order to determine whether screening occurred during or after the MCO period in the state of Kedah. The sociodemographic and clinical factors included age, sex, race, occupation, self-reported presence of medical illness, and smoking status. The MCO period refers to the period when the state of Kedah experienced all the different phases of MCO, which were from 1 January 2021 until 28 June 2021.

Statistical analysis

For data entry and analysis, version 26 of the SPSS software (IBM Inc., Armonk, New York) was used. The data were summarised using frequency counts and percentages for all available data points, as well as the percentages of each stress category (normal, mild, moderate, severe, and very severe). To identify factors associated with stress, both simple and multiple logistic regression analyses were employed. The preliminary variables were chosen based on their p-values (less than 0.25) or their clinical significance, as determined by the simple logistic regression analysis. Using multiple logistic regression, the factors associated with stress were identified. Backward logistic regression (LR) and forward LR approaches were contrasted in order to develop the preliminary main effect model. Multicollinearity and variable interactions were evaluated. Hosmer and Lemeshow's goodness-of-fit test, the classification table, and the receiver operating characteristic (ROC) curve were utilized to evaluate the model's fitness. In determining the factors associated with stress, a p-value of less than 0.05 was considered to be statistically significant.

## Results

Sociodemographic and clinical characteristics of the study population

Table 1 shows the sociodemographic and clinical characteristics of 562 adults in the population of Kedah who were included in the study. The age group of 18 to 34 years old contributed to the majority of the study population, with a total of 367 people (65.3%). The majority of them were female, specifically 391 people (69.6%), and Malays, 536 people (95.4%). Out of the total, 372 participants (66.2%) were employed, while the remaining 190 (33.8%) were unemployed. The percentage of those who answered the screening during MCO was 233 people (41.5%), which was lower compared to the 329 people (58.5%) screened during the non-MCO period. The majority, comprising 362 participants (64.4%), had no known medical illness and were non-smokers, 491 people (87.4%).

**Table 1:** Sociodemographic and clinical characteristics of the adult population in Kedah, Malaysia, during the COVID-19 pandemic (n=562)

Variables	n (%)
Age group (years)	
18 to 34	367 (65.3)
35 to 54	183 (32.6)
55 to 60	12 (2.1)
Sex	
Male	171 (30.4)
Female	391 (69.6)
Race	
Malay	536 (95.4)
Non-Malay	26 (4.6)
Employment status	
Employed	372 (66.2)
Unemployed	190 (33.8)
Movement control order (MCO) period	
Yes	233 (41.5)
No	329 (58.5)
Known medical illness	
Yes	200 (35.6)
No	362 (64.4)
Smoking status	
Yes	71 (12.6)
No	491 (87.4)

Prevalence of stress

A total of 257 participants experienced stress, resulting in a prevalence of 45.7% (95% CI 41.6%, 49.8%). The majority of them did not have stress, consisting of 305 people (54.3%), followed by 69 people (12.3%) who reported severe stress, 67 people (11.9%) who reported moderate, 66 people (11.7%) reported mild, and 55 people (9.8%) reported very severe stress, as shown in Table 2.

**Table 2:** Percentage of stress level among the adult population in Kedah, Malaysia, during the COVID-19 pandemic (n=562)

Stress level	n (%)
Normal	305 (54.3)
		Mild	66 (11.7)
Moderate	67 (11.9)		
		Severe	69 (12.3)
Very Severe	55 (9.8)		

Factors associated with stress

From the univariable analysis, variables with a p-value less than 0.25 included sex, employment status, MCO period, known medical illness, and smoking status. These variables were included in multiple logistic regression analyses. Age was also considered due to its clinical significance (Table 3).

**Table 3:** Factors associated with stress using simple logistic regression analysis (n=562) *simple logistic regression

Variables	Crude OR (95% CI)	Wald statistics (df)	p-value*
Age group (years)			
18 to 34	2.267 (0.671, 7.662)	1.737 (1)	0.188
35 to 54	0.928 (0.269,3.207)	0.014 (1)	0.906
55 to 60	1		
Sex			
Male	1		
Female	3.454 (2.323, 5.134)	37.551 (1)	<0.001
Race			
Malay	1		
Non-Malay	0.731 (0.326, 1.641)	0.576 (1)	0.448
Employment status			
Employed	1		
Unemployed	2.324 (1.626, 3.320)	21.432 (1)	<0.001
Movement control order (MCO) period			
No	1		
Yes	0.422 (0.298, 0.597)	23.650 (1)	<0.001
Known medical illness			
No	1		
Yes	1.227 (0.868,1.734)	1.337 (1)	0.248
Smoking status			
No	1		
Yes	0.607 (0.361,1.019)	3.571 (1)	0.059

The multiple logistic regression analysis revealed that the significant factors were sex, employment status, and MCO period when other variables were controlled. Females had 3.04 times higher odds of experiencing stress compared to males (Adj OR 3.035 95% CI: 2.007 to 4.591, p-value <0.001) when adjusted for employment status and MCO period. Unemployed individuals had 2.17 times higher odds of experiencing stress compared to those employed (Adj OR 2.171 95% CI: 1.480 to 3.185, p-value <0.001) when adjusted for sex and MCO period. Individuals under MCO were 61.7% less likely to experience stress compared to those not under MCO (Adj OR 0.383 95% CI: 0.264 to 0.555, p-value <0.001) when adjusted for sex and employment status (Table 4).

**Table 4:** Final model of factors associated with stress among the adult population in Kedah, Malaysia, during the COVID-19 pandemic (n=562) *multiple logistic regression Constant = -0.848; backward LR method was applied; no multicollinearity and no interaction; Hosmer-Lemeshow test, p-value = 0.645; classification table 67.1% correctly classified; area under receiver operating characteristic (ROC) curve was 70.4%

Variables	B	Adjusted OR (95% CI)	Wald statistics (df)	p-value*
Sex				
Male		1		
Female	1.11	3.035 (2.007, 4.591)	27.677 (1)	<0.001
Employment status				
Employed		1		
Unemployed	0.775	2.171 (1.480, 3.185)	15.704 (1)	<0.001
Movement control order (MCO) period				
No		1		
Yes	-0.961	0.383 (0.264, 0.555)	25.717 (1)	<0.001

## Discussion

Prevalence of stress among the adult population in Kedah, Malaysia, during the COVID-19 pandemic

The prevalence of stress among this population was 45.7%, with a total of 257 people. Among all the categories of stress, severe stress showed the highest percentage (12.3%), which consists of 69 people. The findings of this study are much higher compared to the findings from a study conducted in Malaysia during the initial MCO, which was a 26.5% prevalence of stress. Their study also showed that moderate stress was the highest category (8.9%) [15]. In another study conducted in Malaysia, the percentage of stress was 30.6%, which was also lower than the prevalence reported in the current study [5]. In contrast, another study conducted in Malaysia during the first phase of MCO revealed a higher prevalence of stress (70.0%) than the current study. However, mild stress showed the highest percentage in comparison with the other categories, which was 51.5% [16].

A study performed in Indonesia showed that the prevalence of stress was 25.5%, which was lower compared to the finding in this study, and moderate stress has the highest percentage (10.2%) [17]. The prevalence of stress in the current study is also higher than the reported prevalence in China (24.0%) [18]. The current study was conducted between January 2021 and March 2022, a year after the COVID-19 pandemic was declared. The high percentage of stress, particularly severe stress, can be attributed to the pandemic's unpredictability. The uncertainty about the future months is causing stress. In fact, fifty percent of individuals have reported that the pandemic has made future planning seem impossible [19]. After enduring a year of fear, grief, and the daily chaos caused by the pandemic, including canceled plans, financial difficulties, and missed milestones, the prolonged presence of COVID-19 adds another layer of complexity to what the population had hoped would be a post-pandemic life. Planning becomes challenging in this state of uncertainty, where crucial information about disease severity, quarantine duration, and vaccine efficacy remains difficult to obtain. This applies to planning events, going back to work, and traveling [19].

The timing of COVID-19 phases can have a significant impact on the level of stress experienced by individuals and communities. Depending on the phase of the pandemic, the stressors and challenges faced by individuals and communities may differ, leading to different levels of stress, which could explain the very high stress level (70.0%), in which the mild category (51.5%) predominated in Parveen's study [16]. Since their study was conducted in the first phase of MCO, the early stages of the pandemic, when COVID-19 was first identified and began to spread globally, individuals may have experienced stress related to uncertainty and fear of the unknown. This may have been compounded by a lack of information and conflicting messaging from authorities [20]. Even after enduring over a year of stress, restrictions, and personal sacrifices, the fact that things remain uncertain is exactly what can have an impact on people's mental health [21].

The prevalence and severity of stress during the COVID-19 pandemic may have varied from country to country due to a range of factors. It is anticipated that various cultural systems will have different sets of beliefs about illnesses, which will result in diverse ways of stress coping mechanisms [22]. Cultures that emphasize individualism have been associated with increased levels of stress and feelings of isolation [23]. The prevalence of mental health symptoms varied greatly depending on the stage of the outbreak [24]. The rise in mental health problems throughout the pandemic may be considered as part of the ongoing economic and societal effects of the pandemic's progression [5].

Factors associated with stress among the adult population in Kedah, Malaysia, during the COVID-19 pandemic

This study found that females had 3.035 times higher odds of experiencing stress compared to males. These results are consistent with the UK [6] and Malaysian studies [5] conducted previously. Women have a completely different hormonal system, where the hypothalamic-pituitary-adrenal (HPA) axis initiates faster and generates a larger amount of stress hormones [25]. As a result, women are more prone to emotional responses and express their emotions more readily. Women also tend to shoulder a disproportionate burden of caregiving responsibilities within families and communities. The pandemic has intensified these responsibilities as women have faced increased demands related to children and managing household tasks, including their work responsibilities [26].

In addition, those who are unemployed are 1.480 times more likely than those who are employed to have stress when adjusted for sex and MCO period. This result corresponded to the findings in Hong Kong, where unemployment was associated with higher stress levels [27]. Due to unemployment, individuals experience a reduction in income. Those who are the primary breadwinners in their families face an even greater challenge, as they must not only support themselves but also their family members. This circumstance causes significant stress regarding the duration of income loss and the potential future decline in their quality of life [28].

Regarding MCO status, those under MCO had 0.383 times the odds of experiencing stress compared to those not under MCO when adjusted for sex and employment status. The reason why the MCO may appear as a protective factor could be because the study's duration during the MCO period was shorter compared to the period after the MCO. Hence, the sample size during the MCO period might have been smaller than after the MCO period. Another possible factor is there was rising prayer intensity during the COVID-19 outbreak was mostly explained by the increased need for religious coping [22]. Regardless of the type of religious practice, a higher religiosity level was associated with reduced stress levels during the lockdown [22]. Faith and the capacity for religious coping can be viewed as sources of support for individuals confronting fears and worries, particularly when they have unwavering trust in a higher power and demonstrate patience and gratitude in the face of adversity, including grief and anxiety [29]. This could be attributed to a rise in belief in the Creator, which promotes mental health.

The level of stress and social interactions during the pandemic differ significantly by age group depending on personal circumstances and susceptibility [30]. However, in this study, age may not be the most important factor in determining stress levels when other variables, such as MCO period, employment status, and sex, are considered. These variables may play significant roles in determining stress levels during the pandemic. Depending on their methodologies, different statistical analyses may yield varying results. Additionally, age may interact with other variables in ways that were not completely accounted for in this study.

Despite the potential influence of ethnicity on stress levels, the statistical analysis conducted in this study suggests that ethnicity did not have a significant association with stress levels. Since 95.4% of Kedah's adult population is of Malay ethnicity, which is Islamic in religion, the belief about stress teaches that hardships and difficulties are a natural part of life and that it is important to have patience and trust in God during times of stress and adversity. Muslims are encouraged to turn to God in prayer and seek guidance and support from Him during times of stress and difficulty. Religious coping appears to play a significant role in improving mental health and overall life satisfaction, with its benefits extending to individuals of diverse religious backgrounds, including Christians, Muslims, Buddhists, and Hindus, as well as those who identify as nonreligious or nonspiritual [29].

This is the first study to determine the relationship between the MCO period and stress among adults during the COVID-19 pandemic. This can contribute to the development of more effective community-based interventions for safeguarding the mental health of the general public during future pandemics.

Limitations

This study has certain limitations. It is retrospective nature, based on record reviews, and makes determining the direction of causality challenging. In addition, using the DASS-21 questionnaire as a self-report test only measures the level of depression, anxiety, and stress without diagnosing mental health problems or identifying the underlying causes of negative emotional states.

To develop effective strategies, we suggest conducting research that concentrates on stress-reduction techniques that have been demonstrated to be effective, such as cognitive behavioral therapy (CBT) and mindfulness interventions. In addition, there is a need for a more streamlined questionnaire as an improved screening instrument to address the increasing prevalence and severity of stress.

## Conclusions

During the COVID-19 pandemic in Kedah, approximately 257 (45.7%) of adults experienced stress: 69 (12.3%) severe, 67 (11.9%) moderate, 66 (11.7%) mild, and 55 (9.8%) very severe. The prevalence was higher than those reported in many other regional and international studies. The female gender and unemployment were significant factors associated with stress among adults during this time. MCO period appeared to serve as a buffer against stress. These findings are essential for the development of future plans and strategies to reduce stress and enhance the well-being of the adult population during comparable stressful circumstances.
